# Intraoperative Finding of Anomalous Circumflex Artery Origin in the Dissected Right Coronary Ostium

**DOI:** 10.1055/s-0042-1750412

**Published:** 2022-12-20

**Authors:** Makoto Mori, Andrea Amabile, Prashanth Vallabhajosyula

**Affiliations:** 1 Divison of Cardiac Surgery, Yale School of Medicine, New Haven, Connecticut; 2 Yale Aortic Institute, Yale School of Medicine, New Haven, Connecticut

**Keywords:** anomalous, circumflex, aortic, dissection, Bentall root replacement

## Abstract

Anomalous origin of the left circumflex artery is a rare anatomical variant that may present a unique challenge in emergent aortic surgery.

We present the intraoperative finding of an anomalous origin of left circumflex artery (LCx) in an 85-year-old male who presented with acute Type A aortic dissection, complicated by multiorgan malperfusion and ruptured aortic root with avulsed right coronary ostium. No prior left heart catheterization studies were available at the time of the hospital admission, so the coronary anatomy was unknown. Additionally, the patient presented *in extremis* and did not have a contrasted computed tomography scan, so the coronary anatomy was not visualized clearly. The aortic dissection involved the left coronary ostium, which was repaired with sutures and was salvaged as a coronary button. While dissecting the right coronary artery (RCA), it was noted that the LCx had an anomalous origin from the RCA. Therefore, the LCx was deliberately transected off the RCA and a coronary artery bypass graft was performed with saphenous vein graft ( Fig. 1 ). We felt that direct ostial implantation would have produced too much tension to the LCx attachment; a short “Cabrol”-type graft was considered, but given the small caliber of the LCx, we felt a coronary artery bypass graft was a more reliable revascularization approach. Accordingly, a short saphenous vein graft was anastomosed to the proximal LCx, which laid naturally over the anterior neoaorta. The operation was completed with hemiarch replacement under deep hypothermic circulatory arrest, Bentall root replacement with left coronary button reimplantation, and ascending aortic replacement. The postoperative course was uneventful, and the patient remained symptom-free at 12-month follow-up.

**Fig. 1:**
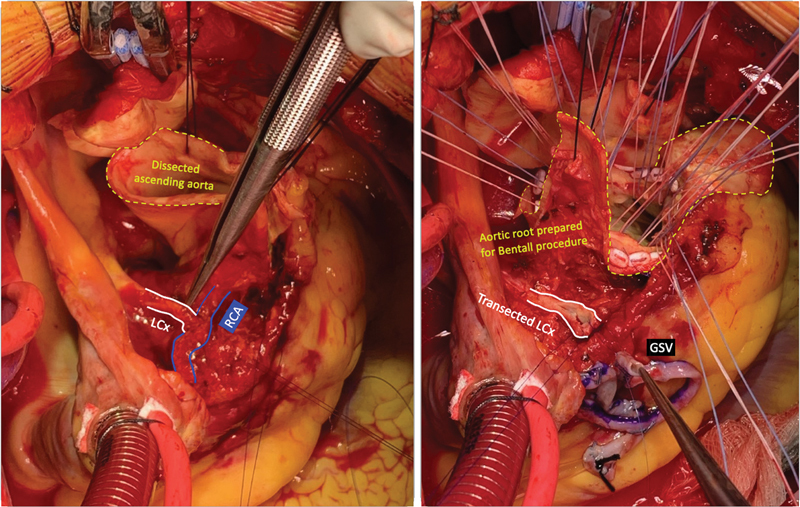
Intraoperative view before (left) and after (right) transection of the anomalous left circumflex artery originating from the right coronary artery. LCx, left circumflex artery; RCA, right coronary artery; GSV, great saphenous vein.

Anomalous origin of the LCx occurs in 0.3% of the population 1 and may present a unique challenge in emergent cardiac surgery. The potential for anomalous coronary origin warrants careful dissection of the aortic root in patients presenting with Type A aortic dissection when the coronary anatomy is unknown. Additionally, careful anatomic evaluation and detailed surgical planning are of extreme importance also in elective operations involving the aortic root when coronary anomalies are suspected.
